# Staff training to reduce behavioral and psychiatric symptoms of dementia in nursing home residents: a systematic review of intervention reproducibility

**DOI:** 10.1590/S1980-57642013DN70300010

**Published:** 2013

**Authors:** Ramon Castro Reis, Débora Dalpai, Analuiza Camozzato

**Affiliations:** 1 Médico Psiquiatra, Mestrando do Programa de Pós Graduação em Ciências da Saúde da Universidade Federal de Ciências da Saúde de Porto Alegre (UFCSPA) Porto Alegre, Brazil.; 2 Estudante de Graduação em Medicina da Universidade Federal de Ciências da Saúde de Porto Alegre (UFCSPA) Porto Alegre, Brazil. Bolsista de Iniciação Científica FAPERGS.; 3 Médica Psiquiatra, Professora de Psiquiatria do Departamento de Clínica Médica da Universidade Federal de Ciências da Saúde de Porto Alegre (UFCSPA) Porto Alegre, Brazil e do Programa de Pós Graduação em Ciências da Saúde da Universidade Federal de Ciências da Saúde de Porto Alegre (UFCSPA) Porto Alegre, Brazil.

**Keywords:** behavioral symptoms, dementia, nursing education, reproducibility of results

## Abstract

Staff training has been cited as an effective intervention to reduce behavioral and psychiatric symptoms of dementia (BPSD) in nursing home residents. However, the reproducibility of interventions can be a barrier to their dissemination. A systematic review of controlled clinical trials on the effectiveness of staff training for reducing BPSD, published between 1990 and 2013 on the EMBASE, PUBMED, LILACS, PSYCHINFO and CINAHL databases, was carried out to evaluate the reproducibility of these interventions by 3 independent raters. The presence of sufficient description of the intervention in each trial to allow its reproduction elsewhere was evaluated. Descriptive analyses were carried out. Despite reference to a detailed procedures manual in the majority of trials, these manuals were not easily accessible, limiting the replication of studies. The professional expertise requirement for training implementation was not clearly described, although most studies involved trainers with moderate to extensive expertise, further limiting training reproducibility.

## Abstract (pt)

Treinamentos de equipes têm sido citados como intervenções efetivas na redução de sintomas comportamentais e psicológicos da demência (SCPD) em residentes de Instituições de Longa Permanência para Idosos. Entretanto, a reprodutibilidade das intervenções pode ser uma barreira para sua disseminação. Uma revisão sistemática de ensaios clínicos controlados da efetividade de treinamento de equipes para a redução dos SCPD publicados entre 1990 e 2013 foi realizada através das bases de dados EMBASE, PUBMED, LILACS, PSYCHINFO e CINAHL para analisar a reprodutibilidade das intervenções por 3 avaliadores independentes. A presença de suficiente descrição da intervenção para permitir sua reprodução foi avaliada. Análise descritiva foi realizada. Apesar da citação na maioria dos estudos de um manual que detalha todos os procedimentos, esses não foram facilmente acessíveis. Embora a experiência profissional requerida não tenha sido claramente mencionada, a maioria dos treinadores tinha moderada a extensa experiência, o que também limita a reprodutibilidade.

## INTRODUCTION

Behavioral and psychiatric symptoms of dementia (BPSD) are highly frequent particularly at moderate to severe stages.^1^ These symptoms are very distressing and represent one of the leading causes of institutionalization of demented subjects.^2^ It has been estimated that BPSD prevalence in patients with dementia living in nursing homes is around 80%.^3,4^ Pharmacological treatment for BPSD has shown poor response,^5^ thus non-pharmacological therapies have a place in this scenario.^6-8^

Systematic reviews have demonstrated that staff training is an effective intervention for reducing BPSD in residents of nursing homes, although methodological weaknesses of trials and the limited number of large-scale studies have been highlighted.^6,8-10^ Concerns about feasibility and reproducibility of staff training have also been raised.^8-10^ A new study using an intervention that has already been applied will only prove feasible if sufficient information about the original procedures is provided in previous studies. It is important to ascertain whether interventions were described in such a way that makes them amenable to replication. Therefore, the aim of this study was to evaluate the provision of well-described operationalization of staff training programs and the presence of guidelines that ensure their reproducibility in future studies.

## METHODS

The process for selecting studies was based on the Cochrane Handbook for Systematic Reviews of Interventions.^11^ Firstly, a systematic literature search was carried out using five databases: EMBASE, PUBMED, LILACS, PSYCHINFO and CINAHL. Three search strategies employing the following keywords were performed: [1] "staff education" OR "staff training" OR "staff development" OR "nursing staff" OR "nursing" AND "dementia" OR "Alzheimer's disease"; [2] "nursing home" OR "care home" AND "caregiver"; [3] "*desenvolvimento de pessoal"* OR "*recursos humanos de enfermagem"* AND "*demência*" OR "*doença de Alzheimer*".

In the second step, titles and abstracts retrieved were reviewed according to the inclusion criteria in order to be selected for full-text revision. These inclusion criteria were: [a] subjects with dementia who presented BPSD as the population of interest; [b] nursing homes or long-term care facilities as the setting; [c] changes in BPSD as the outcome; [d] staff training focusing on dementia care as the intervention; [e] English, Portuguese or Spanish as the publication language; [f] manuscripts published from 1990 to 2013; [g] controlled clinical trials, randomized or otherwise, as the study design. Studies which did not clearly fulfill these inclusion criteria were not retrieved for further revision.

In the third step, a full-text revision was carried out by three independent raters in order to select the studies. For this step, the same inclusion criteria cited above plus an eighth criterion, *i. e*., the presence of training effectiveness in reducing BPSD, were employed.

The presence of a detailed training and procedures description was investigated in the final full-text trials included. Training theoretical framework, intensity of training program (session number, duration and frequency and total duration), presence of individual training, presence of a manual describing all aspects of training, and professional expertise requirement for training implementation were evaluated. Descriptive analyses were performed.

## RESULTS

**Figure 1:**
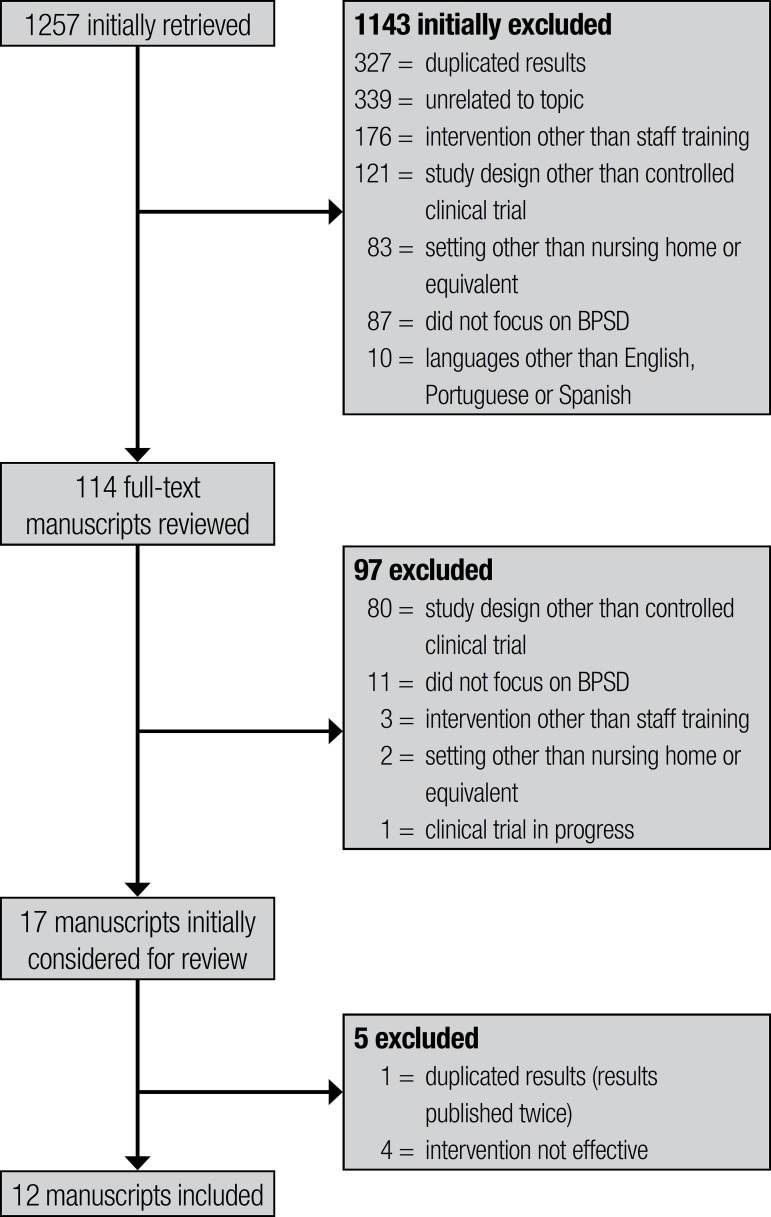
Diagram of retrieved, excluded and included studies.

**Included studies.** The search strategy identified 1257 studies, comprising 327 duplicated studies (same abstract found twice) initially discarded. Abstract screening resulted in the exclusion of 816 studies. The main reasons for exclusion were being unrelated to the topic (339), intervention other than staff training (176) and study design other than controlled clinical trial (121). Of the 114 full-text studies retrieved for detailed inspection, 97 were excluded for not having fulfilled the inclusion criteria. The study design was the most frequent reason for exclusion (80). Seventeen studies were preliminarily considered for review. One out of the 17 had the same results published twice and was therefore excluded.^12^ Additionally, four studies that failed to show staff training effectiveness in reducing BPSD were also excluded,^13-16^ give the main objective was to evaluate the reproducibility of effective interventions. This selection process resulted in 12 studies for final inclusion. Figure 1 contains a detailed diagram of retrieved, excluded and included studies.

**Descriptive analyses of staff training reproducibility.** [a] Theoretical framework of staff training: categories were based on the division proposed by Spector and colleagues,^10^ described as: (i) behavioral-oriented approach with person-environment fit; (ii) communication approach; (iii) person-centered approach; (iv) emotion-oriented approach; and (v) other approaches. The behavioral-oriented approach with person-environment fit was the most frequent framework applied (4 out of 12 studies).^17-20^ This approach takes into account that behaviors are maintained through reinforcement and considers the necessity of adaptation of the environment to suit individual needs. Two studies^21,22^ used person-centered approach (framework focused on individual needs and abilities) and another two trials employed instruction cards to advise on the management of BPSD, showing similar general guidelines but addressing different symptoms.^23,24^ A myriad of other theoretical frameworks were found as singular instances, including communication skills aimed at teaching staff communications strategies to prevent and deal with behavioral problems;^25^ an emotion-oriented approach which helps staff understand and validate the feelings of residents;^26^ abilities-focused care, based on the concept of recovery and maintenance of functional abilities^27^ and a specific approach which uses techniques to reduce the need for restraint.^28^

[b] Intensity of training program: great heterogeneity in the number, frequency and duration of sessions was found. Training programs took from 2 days^25^ to 1 year,^21^ with a minimum of 7^25,27^ to a maximum of 25.5 hours.^23^ Total study duration averaged 4 months, requiring around 15 hours of theoretical and practice exercises.

[c] Detailed procedure description and access (manual detailing all aspects of training, availability of the manual and supplementary material): nine trials provided a brief procedural description in their method section and cited a manual with detailed intervention description and procedures to apply the intervention.^17-20^,^22,23^,^25,27,28^ Two manuals,^29,30^ referred to by Bird et al. and Chenoweth et al., respectively,^19,22^ are freely available only to affiliated members of an American University. Other requests for these documents made to the university library are charged. The manual^31^ cited by Lichtenberg et al.^17^ can be purchased on Amazon's website while a training program with videotapes and a written manual cited by Teri et al.^18^ can be purchased on the University of Washington website.^32^ Deudon et al.^23^ supplied a website address^33^ from which to obtain the complete set of instruction cards (in French) used during their teaching program, to provide caregivers with practical information on what to do and how to respond when faced with BPSD, but it was not possible to retrieve the document from the website. Verkaik et al.^20^ applied an intervention adapted from staff training used in a previous study,^34^ however they did not describe the adaptation procedures. Three trials that had cited manuals detailing procedures^25,27,28^ did not describe how to access the material. One trial^21^ referred readers to two other studies for a more in-depth description.^35,36^ All available manual and supplementary material were published in the English language, except for the set of instruction cards cited by Deudon et al.^23^ which was in French. Senior authors from two studies^17,18^ expressed their availability to resolve doubts concerning the method or to provide handouts and didactic material.

[d] Professional expertise requirement for training implementation: although not all trials indicated the professional expertise requirement for training implementation, most of the studies (9 out of 12) described the expertise of study trainers involved. Nurses,^19,21,22^ psychologists^18,19,24^ and nursing assistants^17,20^ were the professionals who most frequently had implemented the training. Five studies reported moderate to extensive expertise of the trainer conducting the intervention: one study described that the trainers had "geriatric mental health experience"^18^ and in another trial^25^ the person who implemented the intervention had "extensive group leadership experience"; trainers from the study of Chenoweth et al.^22^ had experience of "hundreds of hours of intervention procedures" before its implementation and the trainers of the research carried out by Deudon et al.^23^ were depicted as "professionals with extensive experience of working with residents with dementia".

The characteristics related to the intervention reproducibility of the included studies are summarized in Table 1.

**Table 1:** Characteristics related to intervention reproducibility of the included studies.

	Training description					Detailed proceduresdescription and access		Trainers' expertise
	**Theoretical framework**	**Session number, duration and frequency**	**Total duration**	**Individual sessions**		**Manual detailing all aspects of training**	**Availability of the manual and supplementary material**	
Wells et al. (2000)	Abilities-focused care	14 sessions30 min each1/day to 1/ month	6 months	No		Yes	Not described	Not described
Edberg & Hallberg(2001)	Person-centered approach	12 sessions2h each1/month	12 months	No		Yes	Published(Hallberg & Norberg, 1993)	Not described
Lichtenberg et al. (2005)	Behavioral-oriented approach with person-environment fit	36 sessions20 to 30 min each3/week	3 months	Yes (supervision by the project leader for 1.5 days)		Yes	Published but charged (Lichtenberg et al. 1998); didactic material available from senior author	Trained nursing assistant
Teri et al. (2005)	Behavioral-oriented approach with person-environment fit	2 sessions4h each1/week	2 months	Yes (4 on-site consultations)		Yes	Charged manual and video training obtained by Internet; handouts available from senior author	Clinical psychologist and graduate student in nursing, with geriatric mental health experience
Finnema et al. (2005)	Emotion-oriented approach	2 to 10 sessionsNot described1/week to about 1/month	9 months	No		Not described	Not described	Not described
Robison et al. (2007)	Communication skills	1 session of 4-5h plus 1 session of 2h	2 days	No		Yes	Not described	Extensive group leadership experience
Bird et al. (2007)	Behavioral-oriented approach with person-environment fit	Not described	5 months	Not described		Yes	Published but charged (Bird et al, 2002)	Community registered nurse and clinical psychologist
Chenoweth et al. (2009)	Person-centered approach	6 sessionsNot fully described Not described	4 months	Yes (2 sessions plus regular telephone contact)		Yes	Published but charged (Loveday & Kitwood,1998)	Nurse with hundreds of hours of intervention procedures experience
Deudon et al. (2009)	Practice-based approach	1 session1.5h	2 months	Yes (2 h twice a week during the first month and then once a week during the second month)		Yes	Internet site described, but not possible to retrieve	Professional with extensive experience of working with residents with dementia
Testad et al. (2010)	Skills to reduce the need for restraint approach	1 session of 6h plus6 sessions of 1h1/month	7 months	No		Yes	Not described	Not described
Verkaik et al. (2011)	Behavioral-oriented approach with person-environment fit	3 sessions3h eachAbout 1/month	11 weeks	No		Yes	Not described	Certified nursing assistants
Leone et al. (2012)	Practice-based approach	4 sessions4h each1/week	1 month	No		Not described	Not described	Psychologist

## DISCUSSION

We carried out a systematic review to evaluate the reproducibility of staff training as an effective intervention for BPSD in nursing home residents. Issues concerning theoretical framework, intensity of training program, presence and availability of detailed procedural description and professional expertise requirement for training implementation were evaluated. We concluded that despite references to detailed procedures manuals in the majority of trials, these manuals were not easily accessible, limiting the replication of the studies. Furthermore, the professional expertise requirement for training implementation was not clearly stated, although the trainers who implemented the programs in most studies had moderate to extensive expertise where this finding also limits training reproducibility.

Some important points should be discussed in relation to the presence of detailed description of procedures used in the trials and regarding their accessibility. None of the 12 trials provided sufficient procedure descriptions and none of the manuals cited in the nine studies were accessible free of charge. Additionally, most of the manuals are only published in English language. These facts can hamper intervention replication in countries with different languages and lower socioeconomic levels. Furthermore, each research group applied a specific procedure, although some of them used similar theoretical frameworks. This lack of training standardization prevents broader use and generalization of the interventions.

The training programs were based on approaches ranging from inductive practices ("do this" and "don"t do that")^23,24^ to models based on observed behaviors.^17-20^ They also employed practices that require the identification of communication problems,^25^ emotional comprehension^26^ and personalized care with the elderly.^21,22^ Clearly, higher approach complexity demands greater expertise from the trainers. Although it was not clearly specified what professional expertise is required to implement the training, the programs were conducted by professionals with moderate to extensive experience in the theoretical model and training. This aspect should be considered because the more expertise required, the less feasible and replicable the intervention.

In terms of the number of sessions and total time involved in the programs, these could all be replicated. Nevertheless, the large-scale adoption of training programs with higher intensity requires more planning and organization. We did not evaluate whether the requirements of institutional organization for training implementation were cited in the trials and this represents a limitation of our review. The non-consideration of organizational and system factors in long-term care facilities when planning and implementing training initiatives has been cited as one aspect responsible for difficulties in the sustained transfer of knowledge to practice in staff training programs.^37^

Behavioral and psychological symptoms of dementia are challenging, distressing and very frequent in nursing home residents. More effective therapies for these symptoms are still needed. Staff training programs appear to be a good option in this scenario, but require more standardization. The extent to which a specific training can be repeated, i.e., its reproducibility, is a crucial requirement to carry out a proper evaluation of its effectiveness. Considering results obtained from this review, we believe that concerted efforts should be made to develop a universal, feasible, standardized and easily accessed training program that can be implemented and evaluated worldwide in different cultures and countries.
